# ADAMTS19 Suppresses Cell Migration and Invasion by Targeting S100A16 via the NF-κB Pathway in Human Gastric Cancer

**DOI:** 10.3390/biom11040561

**Published:** 2021-04-12

**Authors:** Yingming Jiang, Xihu Yu, Yandong Zhao, Jintuan Huang, Tuoyang Li, Hao Chen, Junyi Zhou, Zhenze Huang, Zuli Yang

**Affiliations:** 1 Department of Gastrointestinal Surgery, the Sixth Affiliated Hospital, Sun Yat-sen University, Guangzhou 510275, China; jiangym6@mail2.sysu.edu.cn (Y.J.); yuxihu@mail2.sysu.edu.cn (X.Y.); huangjt28@mail.sysu.edu.cn (J.H.); lity35@mail2.sysu.edu.cn (T.L.); chenhao29@mail.sysu.edu.cn (H.C.); zhjy2@mail2.sysu.edu.cn (J.Z.); huangzhz5@mail2.sysu.edu.cn (Z.H.); 2 Guangdong Provincial Key Laboratory of Colorectal and Pelvic Floor Diseases, the Sixth Affiliated Hospital, Sun Yat-sen University, Guangzhou 510275, China; zhaoyd6@mail.sysu.edu.cn; 3 Department of Pathology, the Sixth Affiliated Hospital, Sun Yat-sen University, Guangzhou 510275, China

**Keywords:** ADAMTS19, gastric cancer, migration, invasion, S100A16, NF-κB

## Abstract

A Disintegrin and Metalloproteinase with Thrombospondin motifs 19 (ADAMTS19) has been reported to participate in the pathogenesis of solid cancers. However, its role in gastric cancer (GC) remains undocumented. Using immunohistochemistry (IHC) staining and quantitative real-time polymerase chain reaction (qRT-PCR) on GC tissues and adjacent normal tissues, we found that ADAMTS19 was downregulated in GC tissues (IHC: *p* < 0.001; qRT-PCR: *p* = 0.017). Further investigation revealed that ADAMTS19 correlated with distant metastasis (*p* = 0.008) and perineural invasion (*p* = 0.018) and that patients with low ADAMTS19 had worse overall survival (*p* = 0.021). Gain- and loss-of-function assays showed that ADAMTS19 suppressed cell migration and invasion in vitro. Using bioinformatics analysis and co-immunoprecipitation, immunofluorescence, and dual-luciferase reporter gene assays, we confirmed that ADAMTS19 binds with cytoplasm P65, decreasing the nucleus phosphorylation of P65, a crucial transcription factor in the nuclear factor kappa-B (NF-κB) pathway, thereby downregulating S100 calcium-binding protein A16 (S100A16) expression. S100A16 acted as the downstream of ADAMTS19, reversing the suppression of cell migration and invasion by ADAMTS19 in vitro. A combination of ADAMTS19 and S100A16 expression provided the optimal prognostic indicator for GC. Patients with ADAMTS19^high^-S100A16^low^ had better overall survival than ADAMTS19^low^-S100A16^high^ patients (*p* = 0.006). These results suggest that ADAMTS19 suppresses cell migration and invasion by targeting S100A16 via the NF-κB pathway and that ADAMTS19 and S100A16 are potential metastasis and survival biomarkers for GC.

## 1. Introduction

Gastric cancer (GC) is the fifth most frequently diagnosed cancer and the third leading cause of cancer-related deaths worldwide, with over 1,000,000 new gastric cancer cases in 2018 and an estimated 783,000 related deaths [1,2]. Radical surgery and chemotherapy remain the main treatments. Although screening for early GC has improved overall survival rates, the prognosis of GC remains poor compared to that of other solid tumors [3] because most GC patients are still diagnosed at an advanced stage with either regional, distant or both, metastasis [2], which undermines treatment effectiveness. Hence, it is necessary to reveal the molecular mechanisms underlying the carcinogenesis and progression of GC and find suitable metastasis predictors or therapeutic targets to improve its prognosis.

The A Disintegrin and Metalloproteinase with Thrombospondin motifs (ADAMTS) family comprises 19 members [4]. Aberrant expression or function of ADAMTS family members has been associated with tumor biology [4,5,6,7]. Members of the ADAMTS family, such as ADAMTS2, ADAMTS5, ADAMTS12, and ADAMTS15, can act as cancer suppressors or promoters [8,9,10,11]. Hypermethylation of the ADAMTS19 gene has been observed in gastrointestinal cancers, and epigenetic inactivation of ADAMTS19 can promote metastatic spread in colorectal cancer [12]. However, the role and function of ADAMTS19 in GC remains undocumented.

S100 calcium-binding protein A16 (S100A16) is a member of the EF-hand Ca2 +-binding proteins and participates in tumorigenesis [13,14]. In breast cancer, S100A16 promotes epithelial-mesenchymal transition (EMT) via the Notch1 pathway and correlates with poor prognosis [15]. In pancreatic cancer, it promotes metastasis and progression through the FGF19-mediated AKT and ERK1/2 pathways [16]. In GC, it also acts as a target gene regulating proliferation, invasion, and EMT [17]. However, no studies have investigated the correlation of S100A16 with clinicopathological characteristics and prognosis in GC.

In this study, we focused on the correlation of ADAMTS19 expression with clinicopathological characteristics and overall survival (OS); meanwhile, the mechanisms of ADAMTS19 in GC progression were studied at the molecular level by in vitro assays.

## 2. Materials and Methods

### 2.1. Patients and Cancer Tissue Samples

In our previous study [10], we obtained 176 primary cancer tissue samples collected in the Sixth Affiliated Hospital of Sun Yat-Sen University, Guangzhou, China, from December 2007 to March 2012. Tissue specimens were constructed using tissue microarrays (TMAs) for immunohistochemistry (IHC), the correlations between ADAMTS19 and S100A16 and clinicopathological characteristics were analyzed based on IHC. The patients were followed up until death or until December 31, 2018. Patients lost to follow-up were excluded from the analysis. The interval between the date of surgery and the date of death or last follow-up visit was considered OS. The American Joint Committee on Cancer Staging System (7th edition) was used for GC staging. A group comprising 53 pairs of primary gastric cancerous and adjacent normal mucous tissues (collected in the Sixth Affiliated Hospital of Sun Yat-Sen University, Guangzhou, China, from August 2018 to December 2018) was analyzed immunohistochemically to evaluate the differential expression of ADAMTS19. Another group comprising 24 pairs of primary gastric cancerous and adjacent normal mucous tissues (collected in the Sixth Affiliated Hospital of Sun Yat-Sen University, Guangzhou, China, from March 2019 to August 2018) was used to evaluate the differential expression of S100A16. Informed consent was obtained from all patients. The study was approved by the Research Ethics Committee of Sun Yat-Sen University and complied with the principles of the Declaration of Helsinki.

### 2.2. Immunohistochemistry

We used a biotin-streptavidin horseradish peroxidase (HRP) detection system (ZSGB Bio, Beijin, China) for IHC staining as previously described [10]. In brief, primary rabbit antibodies against ADAMTS19 (ab190073, Abcam, Cambridge, UK, 1:1000) and S100A16 (11456-1-AP, Proteintech, Wuhan, China, 1:1200) were incubated with specimens at 4 °C overnight. The specimens were then incubated with secondary antibodies. Finally, the specimens (TMAs) were developed with diaminobenzidine and counterstained with hematoxylin. A primary antibody diluent was used as a negative control. The ADAMTS19 and S100A16 expression scores were assigned by two pathologists independently. We subsequently used X-tile (Version 3.6.1, Rimm Lab, Connecticut, New Haven, CT, USA) software to select the best cutoff score (5.5 for ADAMTS19 and 6.0 for S100A16) based on a previous study [18]. A group comprising 53 pairs of primary gastric cancerous and adjacent normal mucous tissues was analyzed immunohistochemically to evaluate the differential expression of ADAMTS19. Another group comprising 24 pairs of primary gastric cancerous and adjacent normal mucous tissues was used to evaluate the differential expression of S100A16. The TMAs that contained the 176 primary cancer tissue specimens were used to evaluate the expression of ADAMTS19 and S100A16 and their correlations with clinicopathological characteristics and prognosis in GC. Meanwhile, according to the cutoff scores of ADAMTS19 and S100A16, we divided 176 patients into four groups: ADAMTS19^high^-S100A16^low^ (ADAMTS19 score ≥ 5.5 and S100A16 score < 6.0), ADAMTS19^high^-S100A16^high^ (ADAMTS19 score ≥ 5.5 and S100A16 score ≥ 6.0), ADAMTS19^low^-S100A16^low^ (ADAMTS19 score < 5.5 and S100A16 score < 6.0) and ADAMTS19^low^-S100A16^high^ (ADAMTS19 score < 5.5 and S100A16 score ≥ 6.0); and the OS of the four groups was also evaluated.

### 2.3. Public Online Databases and Related Analyses

The public database Oncomine (https://www.oncomine.org/resource/main.html, access date: 25 September 2018) was used to search for the differential expression of ADAMTS19 in GC and normal tissues. The correlation analysis between S100A16 and P65 was based on GEPIA 2 (http://gepia2.cancer-pku.cn/#index, access date: 12 June 2020). The MEXPRESS database (http://mexpress.be, access date: 24 March 2020) was used to analyze the correlation between ADAMTS19 expression and promoter methylation.

### 2.4. Cell Lines and Culture

Seven human gastric cancer (GC) cell lines (MKN1, MKN45, MGC803, BGC823, HGC27, SGC7901, and AGS) and one human normal gastric mucosal cell (GES1) used in this study were obtained from certified and authenticated cell banks. Detailed source information is as follows: HGC27 (accession number: SCSP-5263), AGS (accession number: SCSP-5262) and SGC7901 (accession number: TCHu46) were obtained from the National Collection of Authenticated Cell Culture; MGC803 (accession number: CBP60485), BGC823 (accession number: CBP60477) and MKN1 (accession number: CBP60486) were obtained from ATCC; MKN45 (accession number: CVCL_0434) was obtained from JCRB Cell Bank. The human normal gastric mucosal cell GES1 was obtained from the Type Culture Collection Cell Bank of the Chinese Academy of Sciences Committee (Shanghai, China). AGS was cultured in DMEM/F12, whereas the other cell lines were cultured in an RPMI 1640 medium. All media were supplemented with 10% fetal bovine serum. The cells were cultured at 37 °C in a humidified atmosphere containing 5% CO_2_.

### 2.5. Plasmid Construction and Transfection

Full-length cDNA encoding ADAMTS19 was amplified using polymerase chain reaction (PCR) from the complete open reading frame (ORF) of ADAMTS19 (NM_133638.4). Then, it was cloned into a pCDH-CMV-MCS-EF1-CopGFP-T2A-Puro (PCHD) vector between the NheI and EcoRI sites. Additionally, two oligonucleotides (5-GGAATTCTCACTTGTCATCGTCGTCCTTGTAGTCACTCTTCTGCTGCAG-3 and 5-CGGCTAGCATGCGCCTGACTCACATC-3) were used to introduce a FLAG epitope at the EcoRI site. Finally, a PCDH-ADAMTS19 plasmid was constructed successfully. Another plasmid, pCDNA3.1-ADAMTS19-3xFlag, was constructed using the same method. The recombinant vectors were transformed into competent *Escherichia coli* DH5α cells (Takara, Kyoto, Japan) for amplification. The identity of the recombinant vectors was confirmed by sequencing. Virus packaging was performed in human embryonic kidney epithelial cell line 293T cells using a Lipofectamine 3000 reagent (Invitrogen, Carlsbad, CA, USA) according to the manufacturer’s instructions. Briefly, PCDH-ADAMTS19 were co-transfected with pCMV-Δ8.91 and pCMV-VSVG plasmids into the 293T cells. Virus supernatants were harvested 48 and 72 h after co-transfection, and the virus titers were determined. For infection, a lentiviral suspension containing approximately 4 × 10^6^ lentiviral particles (multiplicity of infection = 10) and polybrene (5 µg/mL) were added to MGC803 and MKN45 cells. The infected cells were screened by puromycin (Sangon Biotech, Shanghai, China). Puromycin-resistant single-cell clones stably expressing ADAMTS19 were established and verified by quantitative real-time PCR (qRT-PCR) and Western blotting. For ADAMTS19 knockdown, ADAMTS19-shRNA lentiviral and control shRNA vectors were purchased from Genechem (Shanghai, China). The targeting sequence of ADAMTS19-shRNA was 5-GGATGCAGCTATACTTATA-3. BGC823 and SGC7901 cells were also infected using the abovementioned packaged lentiviral vector.

To construct the plasmid of S100A16 and P65, full-length cDNA was amplified from the complete ORF of S100A16 (NM_080388.3) and P65 (NC_000011.10) using PCR. Then, it was cloned into pCDNA3.1 vector, and the recombinant vectors were confirmed by sequencing. Transient transfection was completed using a Lipofectamine 3000 reagent according to the manufacturer’s instructions. Small interfering RNA (siRNA) of S100A16 (RiboBio, Guangzhou, China) was transfected into cells using a Lipofectamine RNAiMAX reagent (Invitrogen, Carlsbad, USA). The siRNA-targeted sequences were as follows: siS100A16-1, 5-GCGAGATGCTCCAGAAAGA-3 (forward); siS100A16-2, 5-GAACCTGGATGCCAATCAT-3 (forward).

### 2.6. RNA Extraction and qRT-PCR

A TRIzol reagent (Invitrogen, Carlsbad, USA) was used to extract total RNA from tissues according to the manufacturer’s instructions, and an RNA-Quick Purification Kit (ES-RN001; Yishan Biotechnology, Shanghai, China) was used to extract total RNA from cells according to the manufacturer’s instructions. Reverse transcription PCR and qRT-PCR were performed as previously described [18]. For the assessment of ADAMTS19, the messenger RNA (mRNA) levels in GC were evaluated using 45 pairs of fresh-frozen primary cancerous and adjacent normal tissues. For the assessment of S100A16, the mRNA levels in GC were evaluated using 80 pairs of fresh-frozen primary cancerous and adjacent normal tissues.

The qRT-PCR primer sequences were as follows: ADAMTS19, 5-AGGCCAGTAACTGCTTGCTAC-3 (forward) and 5-GTCTAGCTTGGTTCTGCATTCTT-3 (reverse); GAPDH, 5-GACAGTCAGCCGCATCTTCTT-3 (forward) and 5-AATCCGTTGACTCCGACCTTC-3 (reverse); S100A16, 5-GCTGTCGGACACAGGGAAC-3 (forward) and 5-TGATGCCGCCTATCAAGGTC-3 (reverse).

### 2.7. Western Blot Analysis

Cell protein was extracted using a T-PER Tissue Protein Extraction Reagent (Thermo Fisher Scientific, Massachusetts, MA, USA) and supplemented with protease and phosphatase inhibitors (ApexBio, Houston, TX, USA) according to the manufacturer’s instructions. The protein concentration was measured using a BCA protein assay kit (Beyotime, Shanghai, China) according to the manufacturer’s instructions. Briefly, lysates containing 40 μg of protein were separated by SDS-PAGE and then transferred to polyvinylidene fluoride membranes (Millipore, Massachusetts, MA, USA). The membranes were then blocked with 5% skimmed milk or 5% bovine serum albumin (BSA) at room temperature for 1 h and incubated with primary antibodies at 4 °C overnight. They were subsequently incubated with corresponding HRP-conjugated secondary antibodies (goat anti-mouse IgG: SA00001-1, 100 μL, Proteintech, 1:10,000; mouse anti-rabbit IgG light chain specific: SA00001-7L, 100 μL, Proteintech; 1:10,000) at room temperature for 1 h. The bands were then detected with an enhanced chemiluminescence reagent and were observed using a ChemiDoc Touch Imaging System (Bio-Rad, California, CA, USA). The bands were quantified using ImageJ (version 1.52v, National Institutes of Health, Bethesda, MD, USA) and normalized with glyceraldehyde-3-phosphate dehydrogenase (GAPDH). Membranes were incubated with the following primary antibodies: ADAMTS19 (ab190073, Abcam, Cambridge, UK, 1:1000), S100A16 (11456-1-AP, Proteintech, Wuhan, China, 1:1000), P65 (#8242, Cell Signaling Technology, Massachusetts, MA, USA, 1:1000), Phospho-P65 (#3033, Cell Signaling Technology, Massachusetts, USA, 1:1000), E-cadherin (20874-1-AP, Proteintech, Wuhan, China, 1:1000), β-catenin (17565-1-AP, Proteintech, Wuhan, China, 1:1000), GAPDH (60004-1-Ig, Proteintech, Wuhan, China, 1:10,000), and PCNA (10205-2-AP, Proteintech, Wuhan, China, 1:2000).

### 2.8. Migration and Invasion Assay

Transwell chambers (#353097, Falcon, New York, NY, USA) with and without Matrigel (#356234, Corning, New York, NY, USA) were used to assess GC cell migration and invasion. In brief, 4 × 10^4^ cells were transferred into the upper chamber with 100 μL of serum-free RMPI-1640, whereas the lower chamber was filled with 700 μL of 10% serum RMPI-1640 to generate a chemoattractant. The cells in the lower chamber were fixed using 4% paraformaldehyde after adequate culturing, and crystal violet was used to stain the cells on the lower membrane surface. Five random fields of the lower membrane surface were photographed using a microscope (Olympus, Tokyo, Japan), and the numbers of migration and invasion cells were calculated using ImageJ. The assays were performed at least in triplicate.

### 2.9. Wound Healing Assay

A sterile tip of a 200-μL pipette was used to make a wound after seeding 1 × 10^6^ cells in a 12-well plate. The cells were then cultured with 1% serum RMPI-1640 for up to six days. An IncuCyte ZOOM system (Essen BioScience, Michigan, MI, USA) was used to capture images of the wound area every 2 h during culturing. Finally, the initial wound area was defined 100%, and ImageJ was used to calculate the percentage of wound closure at the end of the culturing process. The assays were performed at least in triplicate.

### 2.10. RNA Sequencing Array and Bioinformatics Analysis

Whole-transcript deep sequencing (RNAseq) was performed on a BGISEQ-500 platform (BGI, Shenzhen, China). The analysis was designed with two groups of paired MGC803-Vector/ADAMTS19 cells. Heat map analysis of the altered genes was performed using OmicShare Heatmap tools (http://www.omicshare.com/tools, access date: 3 July 2020). The data were also analyzed using the Gene Ontology (GO) method with the free online analysis tool, Database for Annotation, Visualization and Integrated Discovery (DAVID; https://david.ncifcrf.gov/, access date: 3 July 2020) [19,20]. The results of the GO analysis were displayed using the online analysis tool, SangerBox (http://sangerbox.com/Index, access date: 23 December 2020). Gene set enrichment analysis (GSEA) was performed using a GSEA preranked tool, R (version 4.0.3, The R Foundation, New York, NY, USA), and RStudio (1.4.1103, RStudio, Boston, MA, USA).

### 2.11. Dual-Luciferase Reporter Assay

After constructing a pGL4-S100A16 plasmid that contained 1000 bp upstream of the S100A16 promoter region, the identity of the recombinant vector was confirmed by sequencing. The pGL4-S100A16, pRL-TK, pCDNA3.1-ADAMTS19-3xFlag, and pCDNA3.1-P65 plasmids were co-transfected into MGC803 and BGC823 cells using Lipofectamine 3000 according to the manufacturer’s instructions. After culturing for 24–48 h, luciferase activity was measured using a Dual-Luciferase Reporter Gene Assay System (Promega, Wisconsin, WI, USA) according to the manufacturer’s instructions. The assays were performed in triplicate.

### 2.12. Immunofluorescence Assay

MKN45 and MGC803 cells were fixed in 4% paraformaldehyde solution after being transfected with pCDNA3.1-ADAMTS19-3xFlag using a Lipofectamine 3000 reagent and cultured for 48–72 h. The cell membranes were penetrated with a 0.25% Triton X100 (meilunbio, Dalian, China) solution. After 15 min, 1% BSA was transferred to block the cells at room temperature for 30 min. Primary antibodies (Flag-tag: 390002, ZenBio, Sicuan, China, 1:1000; P65: 10745-1-AP, Proteintech, Wuhan, China, 1:200) were incubated at 4 °C for 12–16 h. Primary antibody binding and nuclei were observed using fluorescence secondary antibodies (anti-rabbit: A11034, Invitrogen, 1:200; anti-mouse IgG: A11003, Invitrogen, 1:200) and DAPI (4’,6-diamidino-2-phenylindole; #C1002, Beyotime, Shanghai, China, 1:1000) staining, respectively. Finally, the transfected MKN45 and MGC803 cells were observed and photographed using a confocal microscope (Leica, Weztlar, Germany).

### 2.13. Nuclear and Cytoplasmic Protein Extraction Assay

A nuclear and cytoplasmic protein extraction assay was performed using a NE-PER Nuclear and Cytoplasmic Extraction Reagent Kit (#78833, Thermo Fisher Scientific, Massachusetts, USA) according to the manufacturer’s instructions. In brief, after protein extraction, 20 μg of protein was added into each well of 8% SDS-PAGE. Subsequently, Western blotting was performed as described above.

### 2.14. Co-Immunoprecipitation Assay

Stably overexpressed ADAMTS19 and control MGC803 cells were lysed using Pierce IP Lysis Buffer (#87788, Thermo Fisher Scientific, Massachusetts, USA) according to the manufacturer’s instructions. Cell supernatants were centrifuged at 4 °C, and then 50 μL of a protein A/G agarose bead solution was added to every 100 μL of cell supernatant. ADAMTS19 (ab190073, Abcam, Cambridge, UK) and P65 (#8242, Cell Signaling Technology, Massachusetts, USA) primary antibodies were used to pull down the proteins interacting with ADAMTS19 and P65 at 4 °C overnight. Immunoglobulin G protein was used as a positive control. After protein extraction, detections were performed by Western blotting as described above.

### 2.15. Statistical Analysis

Statistical analysis was performed using IBM SPSS Statistics version 21.0 (IBM, New York, NY, USA). Continuous variables were presented as the means ± standard deviations and analyzed by Student’s t test. Categorical variables were assessed using the chi-squared test, or the Wilcoxon signed-rank test as appropriate. Overall survival was calculated using the Kaplan–Meier method, and comparisons were performed using the log-rank test. Potential prognostic factors for survival were assessed in multivariate analysis using Cox proportional hazards regression following a backward elimination process. The correlation with ADAMTS19 with promoter methylation were assessed using Pearson’s correlation coefficient. A *p*-value of less than 0.05 was considered statistically significant.

## 3. Results

### 3.1. ADAMTS19 Is Downregulated in Human GC

**Figure 1:**
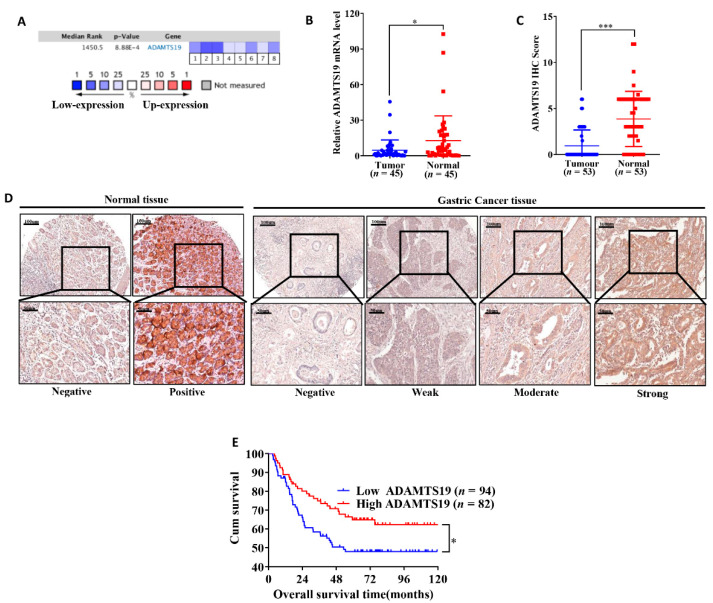
ADAMTS19 is downregulated in gastric cancer (GC). (**A**) Oncomine analysis revealed that ADAMTS19 mRNA expression levels are lower in tumor tissue than in normal tissue by meta-analysis. The intensity gene expression indexed with the color code bars, the median rank is used to demonstrated the gene rank in each analysis. (**B**) Quantitative real-time polymerase chain reaction (qRT-PCR) on 45 paired samples showed that ADAMTS19 mRNA expression levels were lower in tumor tissue than in adjacent normal tissue. (**C**) Immunohistochemistry (IHC) analysis of 53 paired samples showed that ADAMTS19 expression was lower in tumor tissue than in adjacent normal tissue. (**D**) Representative IHC images of tumor and adjacent normal tissue. (**E**) High ADAMTS19 expression predicted better overall survival than low ADAMTS19 expression in GC patients (the best cutoff score of 5.5 was obtained using X-tile). The data are presented as means ± standard deviations. * *p* < 0.05; *** *p* < 0.001.

We used Oncomine and GC specimens from our institution to evaluate the expression of ADAMTS19. The Oncomine analysis revealed that ADAMTS19 mRNA levels were significantly lower in cancer tissues than in adjacent normal tissues (median rank: 1450.5, *p* < 0.001; Figure 1A). Similar results were obtained by qRT-PCR from 45 pairs of cancer and normal tissues (4.67 ± 8.72 vs. 12.76 ± 20.96, *p* = 0.017; Figure 1B) and by IHC from 53 pairs (0.9 ± 1.72 vs. 3.8 ± 3.00, *p* < 0.001; Figure 1C). Representative IHC images are shown in Figure 1D. Pearson’s correlation coefficient showed that ADAMTS19 expression negatively correlates with promoter methylation (Figure S1). Taken together, these results suggest that ADAMTS19 is downregulated in human GC.

### 3.2. ADAMT19 Is Associated with Clinicopathological Characteristics and Survival in GC

We evaluated the correlations between ADAMTS19 and clinicopathological characteristics in 176 GC samples using IHC. ADAMTS19 protein expression was significantly associated with the M stage (Distant Metastasis, *p* = 0.008; Table 1), Lauren’s classification (*p* = 0.007; Table 1), and perineural invasion (*p* = 0.018; Table 1). In contrast, it did not significantly correlate with age, gender, histologic type, differentiation, T stage (Invasion Depth), N stage (Lymph Node Metastasis), TNM stage, and vessel invasion. Patients with high ADAMTS19 expression had significantly better OS than low ADAMTS19 patients (*p* = 0.021; Figure 1E). Univariate analysis identified five prognostic indicators: invasion depth, lymph node metastasis, distant metastasis, vessel invasion, and ADAMTS19 expression. However, in multivariate analysis, ADAMTS19 was not an independent prognostic marker (Table 2). Nevertheless, taken together, the results suggest that ADMTS19 could be a predictor of metastasis and a prognostic marker in GC.

### 3.3. ADAMTS19 Suppresses GC Cell Migration and Invasion

**Figure 2:**
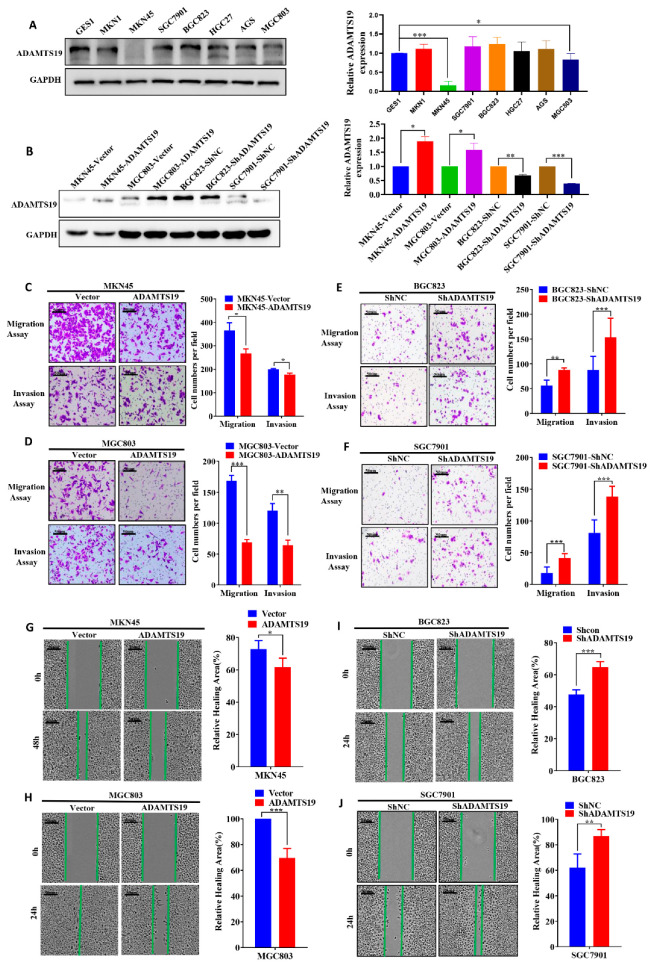
ADAMTS19 suppresses GC cell migration and invasion in vitro. (**A**) ADAMTS19 protein expression levels measured in seven gastric cancer cell lines (MKN1, MKN45, SGC7901, AGS, BGC823, HGC27, and MGC803) and one normal gastric epithelial cell line (GES1) by Western blotting. (**B**) MGC803 and MKN45 cells with low expression of endogenous ADAMTS19 were chosen to construct stably-overexpressed ADAMTS19 cells transfected with a lentiviral vector, and BGC823 and SGC7901 cell lines with high endogenous ADAMTS19 were stably transfected with shRNA through a lentiviral vector. ADAMTS19 expression in stably transfected cells was confirmed by Western blotting. (**C**–**F**) Overexpression of ADAMTS19 suppressed GC cell migration and invasion, while ADAMTS19 knockdown reversed the effects. (**G**–**J**) Wound healing assays showed that overexpression of ADAMTS19 significantly inhibited wound healing in MKN45 and MGC803, whereas ADAMTS19 knockdown promoted wound healing in BGC823 and SGC7901. The data are presented as means ± standard deviations. * *p* < 0.05; ** *p* < 0.01; *** *p* < 0.001.

To reveal the underlying biological function of ADAMTS19 in GC, we used GC cell lines to perform functional assays in vitro. Western blot analysis revealed the expression of ADAMTS19 in GC cell lines (Figure 2A). MGC803 and MKN45 with low expression of endogenous ADAMTS19 were chosen to construct stably overexpressed ADAMTS19 cells transfected with a lentiviral vector (Figure 2B). BGC823 and SGC7901 with high endogenous ADAMTS19 were chosen to construct stably knockdown ADAMTS19 cells transfected with a lentiviral vector (Figure 2B). Transwell assays revealed that ectopic expression of ADAMTS19 inhibited cell migration (356.66 ± 32.47 vs. 267.66 ± 18.93, *p* = 0.011, Figure 2C; 168.67 ± 25.27 vs. 68.89 ± 13.50, *p* < 0.001, Figure 2D). Similarly, invasion assays revealed that ADAMTS19 inhibited cell invasion (200.00 ± 2.82 vs. 177.50 ± 6.36, *p* = 0.045, Figure 2C; 120.44 ± 34.62 vs. 64.33 ± 24.92, *p* = 0.001, Figure 2D). Conversely, as speculated, ADAMTS19 knockdown promoted cell migration (56.33 ± 11.01 vs. 88.00 ± 3.61, *p* = 0.009, Figure 2E; 17.90 ± 9.71 vs. 41.50 ± 7.25, *p* < 0.001 Figure 2F) and invasion (87.80 ± 27.09 vs. 153.44 ± 38.65, *p* < 0.001, Figure 2E; 81.40 ± 20.42 vs. 138.70 ± 15.76, *p* < 0.001, Figure 2F). Wound healing assays revealed that overexpression of ADAMTS19 inhibited wound healing (72.88 ± 5.19 vs. 61.62 ± 5.60, *p* = 0.011, Figure 2G; 100.00 ± 0.00 vs. 69.60 ± 7.39, *p* < 0.001, Figure 2H). Conversely, ADAMTS19 knockdown promoted wound healing (47.59 ± 2.97 vs. 64.71 ± 3.41, *p* < 0.001, Figure 2I; 62.23 ± 10.60 vs. 86.96 ± 4.95, *p* = 0.005, Figure 2J). These results suggest that ADAMTS19 suppresses GC cell migration and invasion.

### 3.4. S100A16 Is a Downstream of ADAMTS19 and Acts as a Tumor Promoter, Promoting Cell Migration and Invasion

**Figure 3:**
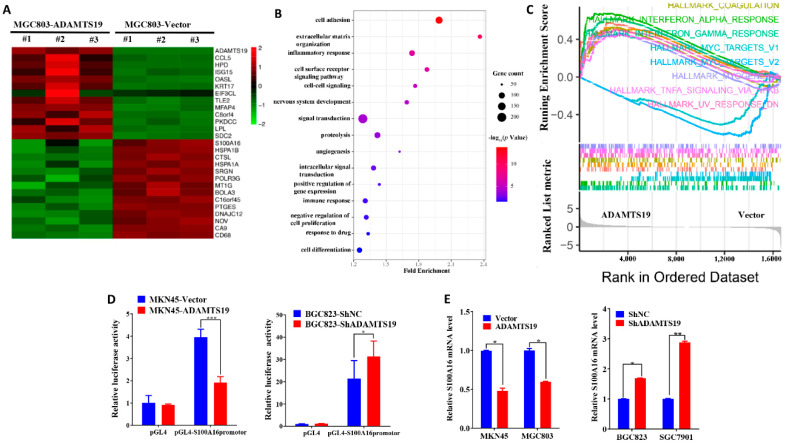

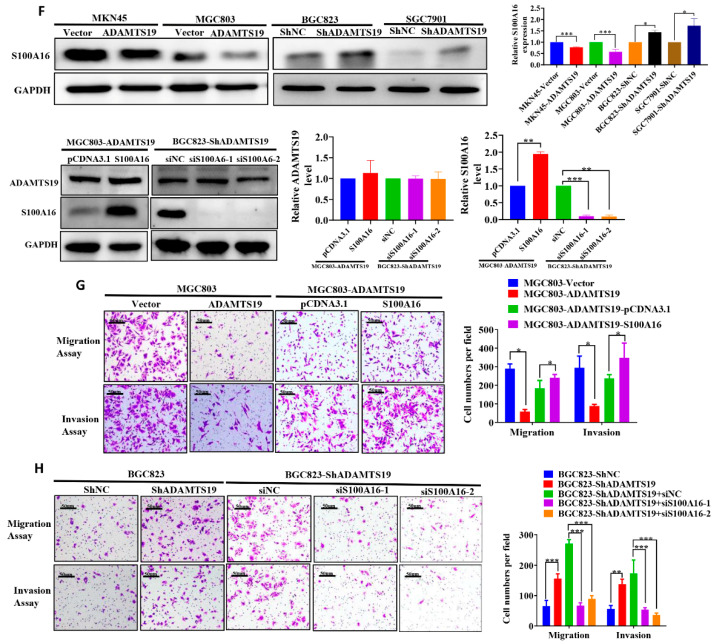
S100A16 is a downstream of ADAMTS19 and acts as a tumor promoter, promoting cell migration and invasion. (**A**) Heat map analysis showed altered genes in MGC803-ADAMTS19 and MGC803-vector cells. (**B**) Gene Ontology analysis showed that the altered genes are involved in biological processes, such as cell adhesion, extracellular matrix organization, inflammatory response, and cell surface receptor signaling pathway. (**C**) Gene set enrichment analysis (GSEA) showed the enrichment of ADAMTS19-associated genes in HALLMARK_TNF_SIGNALING VIA_NFKB and other pathways. The plot was drawn using R and RStudio. (**D**) Dual-luciferase gene reporter assays revealed that ADAMTS19 could regulate the S100A16 transcription directly in MKN45 and BGC823. (**E**,**F**) Quantitative RT-PCR and Western blot analysis showed that S100A16 protein levels were affected by ADAMTS19. (**G**,**H**) Ectopic expression of S100A16 reversed the inhibition of cell migration and invasion by ADAMTS19 in MGC803 and BGC823 cells. The data are presented as means ± standard deviations. * *p* < 0.05; ** *p* < 0.01; *** *p* < 0.001.

We used RNAseq assays and bioinformatics analysis to detect gene expression changes triggered by ADAMTS19 overexpression. Heat map analysis showed distinct genes after ADAMTS19 overexpression (Figure 3A; details on the genes are displayed in Supplementary Table S1). Gene Ontology analysis was performed to evaluate related biological processes of the identified genes based on variability ranking. Consistent with our observations in vitro, the GO analysis showed that ADAMTS19-related genes were mostly involved in pathways such as cell adhesion, extracellular matrix organization, inflammatory response, and cell surface receptor signaling pathway (Figure 3B). Moreover, HALLMARK_TNFA_SIGNALING_VIA_NFKB and other pathways were enriched in GSEA (Figure 3C). Among the dysregulated genes, S100A16 attracted our attention and was confirmed by a dual-luciferase reporter gene assay (Figure 3D). Furthermore, qRT-PCR and Western blot analysis showed that S100A16 expression was affected by ADAMTS19 expression (Figure 3E,F). Conversely, Western blot analysis showed that S100A16 expression did not affect ADAMTS19 expression (Figure 3F). We further investigated the role of S100A16 by functional assays in vitro. Overexpression of S100A16 reversed the suppression of cell migration (183.50 ± 42.63 vs. 240.50 ± 17.54, *p* = 0.048, Figure 3G) and invasion (237.75 ± 19.61 vs. 347.50 ± 80.28, *p* = 0.037, Figure 3G) by ADAMTS19 overexpression. Conversely, S100A16 silencing inhibited migration (siNC: 270.67 ± 13.05 vs. siS100A16-1: 67.00 ± 9.54, *p* < 0.001; siNC: 270.67 ± 13.05 vs. siS100A16-2: 89.33 ± 10.21, *p* < 0.001; Figure 3H) and invasion (siNC:173.00 ± 43.97 vs. siS100A16-1: 54.00 ± 6.08, *p* < 0.001; siNC: 173.00 ± 43.97 vs. siS100A16-2: 36.33 ± 6.03, *p* < 0.001; Figure 3H) promoted by ADAMTS19 knockdown. These findings indicate that ADAMTS19 is the upstream regulator of S100A16.

### 3.5. ADAMTS19 Inhibits Cell Migration and Invasion via the NF-κB/S100A16 Axis

**Figure 4:**
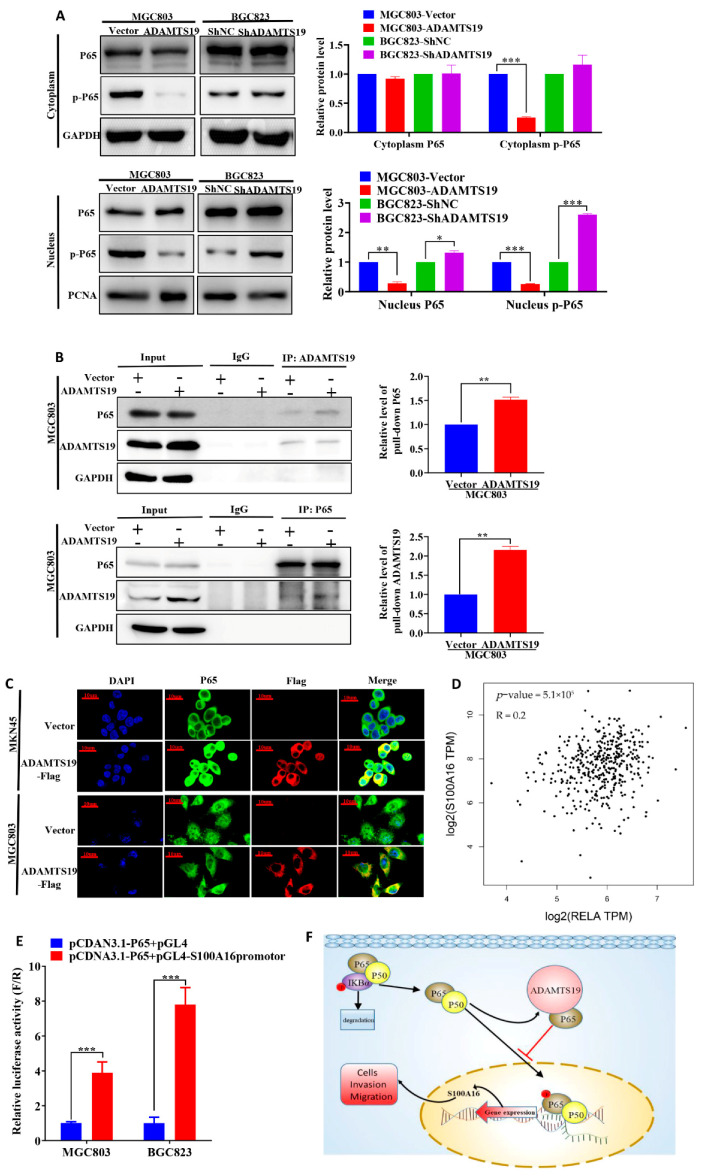
ADAMTS19 downregulates S100A16 by participating in the process of P65 phosphorylation of the NF-κB pathway. (**A**) Nucleus phospho-P65 was decreased in MGC803-ADAMTS19 and increased in BGC823-shADAMTS19 cells. (**B**) Co-immunoprecipitation assays revealed that ADAMTS19 interacted with P65 in MGC803. (**C**) Double immunofluorescence assays revealed that ADAMTS19 was co-localized with P65 in cytoplasm in MGC803 and MKN45 cells after transfection with pCDNA3.1-ADAMTS19-3xFlag, and nucleus P65 was significantly downregulated in MGC803 cells. (**D**) The positive correlation between S100A16 and P65 was verified using GEPIA 2. (**E**) Dual-luciferase reporter gene assays showed that P65 acted as a transcription factor of S100A16. (**F**) Brief schematic model showing that ADAMTS19 regulated S100A16 via the NF-κB pathway and participated in P65 phosphorylation. The data are presented as means ± standard deviations. * *p* < 0.05; ** *p* < 0.01; *** *p* < 0.001.

We further explored the underlying ADAMTS19-S100A16 mechanism. ADAMTS19 overexpression downregulated nucleus phospho-P65, whereas ADAMTS19 knockdown significantly upregulated it (Figure 4A). Co-immunoprecipitation assays revealed that ADAMTS19 could bind with P65 in cytoplasm (Figure 4B). Immunofluorescence assays showed that ADMTS19 was co-localized with P65 in cytoplasm in MKN45 and MGC803 after transfection of the pCDNA3.1-ADAMTS19-3xFlag plasmid (Figure 4C). Correlation analysis powered by GEPIA 2 revealed that S100A16 positively correlated with P65 (*p* < 0.001; Figure 4D). Furthermore, dual-luciferase reporter gene assays showed that P65 promoted the transcriptional activity of S100A16 in MGC803 and BGC823 cells (Figure 4E). These results suggest that ADAMTS19 regulates S100A16 by influencing phospho-P65 of the NF-κB pathway, suppressing cell migration and invasion.

### 3.6. S100A16 Correlates with Clinicopathological Characteristics and Prognosis in GC

**Figure 5:**
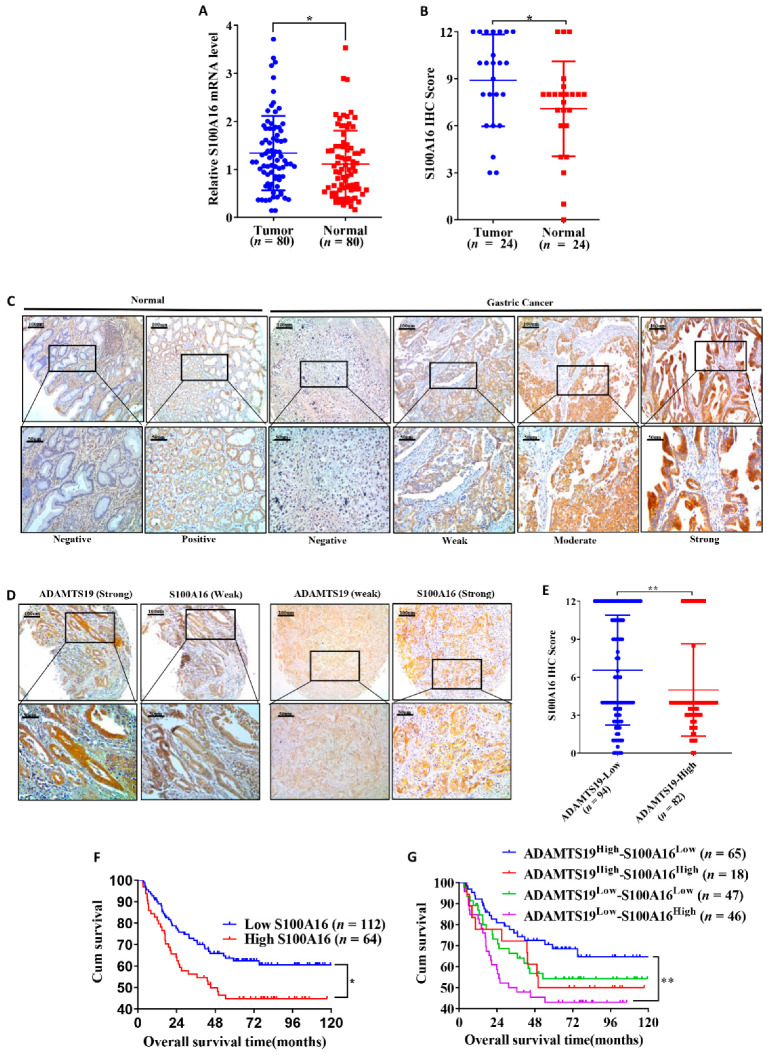
A combination of ADAMTS19 and S100A16 expression predicts the prognosis of GC patients. (**A**) Quantitative RT-PCR on 80 paired GC tissues showed that S100A16 mRNA expression was higher in tumor tissue than in adjacent normal tissue. (**B**) IHC analysis of 24 paired tissues showed that S100A16 expression was higher in tumor tissue than in adjacent normal tissue. (**C**,**D**) Representative IHC images of S100A16 and ADAMTS19 at the same points of tissue microarrays. (**E**) The negative correlation between ADAMTS19 and S100A16 was confirmed by IHC. (**F**) High S100A16 expression predicted poorer overall survival than low expression in GC patients (the best cutoff score of 6.0 was obtained using X-tile). (**G**) Survival analysis layered by the expression of ADAMTS19 and S100A16 in tissue microarrays of GC patients showed that ADAMTS19^high^-S100A16^low^ patients had the best overall survival (OS), whereas ADAMTS19^low^-S100A16^high^ patients had the worst OS. * *p* < 0.05; ** *p* < 0.01.

We further analyzed the expression of S100A16 in cancer tissues and adjacent normal tissues. Quantitative RT-PCR (1.33 ± 0.77 vs. 1.10 ± 0.69, *p* = 0.038; Figure 5A) and IHC (8.89 ± 2.93 vs. 7.08 ± 3.02, *p* = 0.023; Figure 5B) showed that S100A16 expression was higher in cancer than in adjacent normal tissues. Representative IHC images are shown in Figure 5C. Furthermore, we analyzed the correlation of S100A16 with clinicopathological characteristics and prognosis in 176 GC patients. S100A16 significantly correlated with the degree of Lauren’s classification (*p* = 0.020; Table 1) and differentiation (*p* = 0.011; Table 1). In contrast, no significant correlations were found between S100A16 and age, gender, histologic type, perineural invasion, T stage, M stage, N stage, TNM stage, and vessel invasion (Table 1). Representative IHC images are shown in Figure 5D. Further, S100A16 expression negatively correlated with ADAMTS19 expression (6.55 ± 4.33 vs. 4.98 ± 3.65, *p* = 0.001; Figure 5E). Patients with high S100A16 expression had significantly worse OS than low S100A16 patients (*p* = 0.023; Figure 5F). Further investigation revealed that the co-expression of ADAMTS19 and S100A16 correlated with OS, with ADAMTS19^high^-S100A16^low^ patients having better OS than ADAMTS19^low^-S100A16^high^ patients (*p* = 0.006; Figure 5G). Multivariate analysis revealed that S100A16 expression was an independent prognostic marker of GC (*p* = 0.030; Table 2). These results suggest that S10016 is associated with differentiation and is an independent GC prognostic factor.

## 4. Discussion

Members of the ADAMTS family are involved in the progress of solid tumors [5,6]. Members such as ADAMTS2, ADAMTS5, ADAMTS12, and ADAMTS15 act as cancer suppressors or promoters [8,9,10,11]. Moreover, ADAMTS19 is downregulated and correlates with prognosis in colorectal cancer [12]. However, its role in GC has hitherto been unknown. This is the first study to explore its exact role in GC. Using qRT-PCR and IHC, we found that ADAMTS19 is downregulated in cancer tissues compared to adjacent normal tissues. The results were consistent with our Oncomine analysis.

DNA methylation is frequently described as a “silencing” epigenetic marker. Methylation blocks the start of transcription, not elongation [21]. We used the MEXPRESS database to analyze the correlation between ADAMTS19 expression and promoter methylation [22]. In line with a previous study on colorectal cancer [12]. Pearson’s correlation coefficient showed that ADAMTS19 expression negatively correlates with promoter methylation (Figure S1). This result indicates that promoter hypermethylation leads to downregulated ADAMTS19 expression in GC. Hence, we speculate that ADAMTS19 is a tumor inhibitor and plays a crucial role in the pathogenesis of GC.

We also found that ADAMTS19 protein significantly correlates with distant metastasis, Lauren’s classification, differentiation and perineural invasion in GC. Survival analysis revealed that patients with low ADAMTS19 expression have worse OS than high ADAMTS19 patients, which is consistent with the findings of a study on colorectal cancer [12]. Subsequent cell functional assays showed that ADAMTS19 suppresses GC cell migration and invasion. We further performed mechanistic experiments and rescue assays and demonstrated that S100A16 is the downstream of ADAMTS19, acting as a tumor promoter involved in carcinogenesis. This finding is consistent with previous studies on S100A16 [15,23]. In contrast, ADAMTS19 and S100A16 were not associated with EMT significantly, but they tended to affect the levels of E-cadherin and β-catenin measured by Western blot (Figure S2).

Although previous studies have reported that S100A16 tends to act as a tumor promoter [16,23,24], none examined the correlation between S100A16 expression and clinicopathological characteristics of GC. This is the first study to show that high S100A16 expression tends to result in poor differentiation and that patients with high S100A16 expression have worse OS than low S100A16 patients. We also showed that S100A16 is an independent predictor of GC prognosis, with ADAMTS19^high^-S100A16^low^ patients having the best OS and ADAMTS19 ^low^-S100A16 ^high^ patients having the worst OS. In addition, our study reveals that both ADAMTS19 and S100A16 were correlated with Lauren’s classification, but not WHO classification (Table 1). WHO classification and Lauren’s classification are the two main classifications of histologic type of gastric cancer. WHO classification includes not only the common types of gastric cancer, but also the rare and special types. However, WHO classification is not suitable for prognosis analysis of gastric cancer due to its relatively fine classification and great differences in biological behaviors between different types of gastric cancer. The advantages of Lauren’s classification are simplicity, ease of grasping, and high repeatability between different observers. However, the disadvantage of Lauren’s typing is that it is too general to distinguish gastric cancer with different biological behaviors. In our study, there were no significant differences in the WHO classification (Figure S4A) and Lauren’s classification (Figure S4B) of 176 patients. The intestinal type has a trend to obtain better OS than diffuse type and mixed type, the non-significant difference between the groups may be caused by small sample size.

We further explored the regulatory ADAMTS19-S100A16 mechanism. The NF-κB pathway, especially its important member P65, has a complicated relationship with cancer [25,26,27,28]. Shan et al. demonstrated that P65 binds to the mortalin promoter and promotes ovarian cancer cell proliferation and migration via regulating mortalin [26]. Zhang et al. showed that it can directly bind to the promoter of cyclin D1, mediating an increase in the protein’s expression [28]. In this study, we found that ADAMTS19 can bind to cytoplasm P65 and inhibit the nuclear translocation of P65, decreasing nucleus phospho-P65. We also found that P65 can regulate the transcription of S100A16 and positively correlates with S100A16 expression. We thus demonstrated that ADAMTS19 inhibits cell migration and invasion via the NF-κB/S100A16 axis.

To our knowledge, our study is not only the first to reveal the correlation between ADAMTS19 and S100A16 in GC but also the first to explore the correlation of the differential co-expression of ADAMTS19 and S100A16 with the prognosis of GC. Moreover, it is the first study to elucidate the regulatory ADAMTS19-S100A16 mechanism. The role of ADAMTS19 and S100A16 warrants further research. Studies on animal models should be conducted to further explore the role of ADAMTS19 in GC cell migration and invasion. Moreover, future studies should further investigate the specific methylation mechanism of ADAMTS19. Finally, further research is needed to clarify the exact transcriptional mechanism of S100A16 regulated by P65.

## 5. Conclusions

In conclusion, this study demonstrates that ADAMTS19 is downregulated and S100A16 is upregulated in GC. Both ADAMTS19 and S100A16 correlate with clinicopathological characteristics and the prognosis of GC. ADAMTS19^high^-S100A16^low^ patients have the best OS, whereas ADAMTS19^low^-S100A16^high^ patients have the worst OS. Functionally, ADAMTS19 suppresses GC cell migration and invasion in vitro. Mechanistically, ADAMTS19 physically binds to cytoplasm P65, downregulating S100A16, thereby suppressing cell migration and invasion. The clinical and biological significance of the ADAMTS19/NF-κB/S100A16 axis suggests that ADAMTS19 and S100A16 can serve as prognostic biomarkers of GC.

## Figures and Tables

**Table 1:** Correlation between ADAMTS19, S100A16 expression and clinicopathological characteristics of gastric patients (*n* = 176).

Clinicopathological Characteristics	Low ADAMTS19	High ADAMTS19	*p*- Value	Low S100A16	High S100A16	*p*- Value
Number	94	82		112	64	
Age			0.120			0.864
<60 years	50 (53.2)	34 (41.5)		54 (48.2)	30 (46.9)	
≥60 years	44 (46.8)	48 (58.5)		58 (51.8)	34 (53.1)	
Gender			0.224			0.072
Male	62 (65.9)	61 (74.4)		73 (65.2)	50 (78.1)	
Female	32 (34.1)	21 (25.6)		39 (34.8)	14 (21.9)	
Histologic type			0.186			0.672
Tubular or papillary adenocarcinoma	75 (79.8)	73 (89.1)		92 (82.1)	56 (87.5)	
Signet-ring cell carcinoma	13 (13.8)	6 (7.3)		14 (12.5)	5 (7.8)	
Mucinous adenocarcinoma	6 (6.4)	2 (2.4)		5 (4.5)	3 (4.7)	
^a^ Others	0 (0)	1 (1.2)		1 (0.9)	0 (0)	
Differentiation			0.055			0.011
Well-Moderately	76 (80.9)	56 (68.3)		21 (18.8)	23 (35.9)	
Poor	18 (19.1)	26 (31.7)		91 (81.2)	41 (64.1)	
Lauren’s classification			0.007			0.020
Diffuse type	54 (57.5)	28 (34.1)		60 (53.6)	22 (34.4)	
Intestinal type	31 (32.9)	39 (47.6)		36 (32.1)	34 (53.1)	
Mixed type	9 (9.6)	15 (18.3)		16 (14.3)	8 (12.5)	
Invasion Depth			0.077			0.576
T1 + T2	15 (15.9)	22 (26.8)		25 (22.3)	12 (18.8)	
T3 + T4	79 (84.1)	60 (73.2)		87 (77.7)	52 (81.2)	
Lymph Node Metastasis			0.055			0.795
N0	19 (20.2)	27 (32.9)		30 (26.8)	16 (25.0)	
N1/N2/N3	75 (79.3)	55 (67.1)		82 (73.2)	48 (75.0)	
Distant Metastasis			0.008			0.145
M0	72 (76.6)	75 (91.5)		97 (86.6)	50 (78.1)	
M1	22 (23.4)	7 (8.5)		15 (13.4)	14 (21.9)	
TNM Stage			0.110			0.486
I+II	26 (27.7)	32 (39.1)		39 (34.9)	19 (29.7)	
III+IV	68 (72.3)	50 (60.9)		73 (65.1)	45 (70.3)	
Perineural Invasion			0.018			0.493
Absent	36 (38.3)	46 (56.1)		50 (44.6)	32 (50.0)	
Present	58 (61.7)	36 (43.9)		62 (55.4)	32 (50.0)	
Vessel Invasion			0.287			0.327
Absent	51 (54.3)	51 (62,2)		68 (60.7)	34 (53.1)	
Present	43 (45.7)	31 (37.8)		44 (39.3)	30 (46.9)	

Statistical analyses were performed by the Pearson χ2 test; ^a^ Others: hepatoid adenocarcinoma and squamous carcinoma.

**Table 2:** Univariate and multivariate analyses for the prognostic factors of overall survival in gastric cancer patients (*n* = 176).

Variable	Univariate Analysis		Multivariate Analysis	
	HR (95% CI)	*p*-Value	HR (95% CI)	*p*-Value
Age (≥60 years vs. <60 years)	0.812 (0.515–1.281)	0.370	0.589 (0.367–0.964)	0.028
Gender (male vs. female)	0.859 (0.526–1.404)	0.545		
Histology (tubular/papillary adenocarcinoma	1.150 (0.591–2.238)	0.681		
vs. the others)				
Differentiation (well/moderate vs. poor)	0.732 (0.421–1.272)	0.732		
Invasion depth (T3/T4 vs. T1/T2)	7.051 (2.572–19.335)	< 0.001		
Lymph Node Metastasis (N+ vs. N0)	5.690 (2.466–13.130)	<0.001		
Distant metastasis (M1 vs. M0)	3.487 (2.110–5.761)	<0.001	2.333 (1.387–3.925)	0.001
Perineural Invasion (present vs. absent)	3.067 (1.836–5.126)	<0.001	2.539 (1.486–4.340)	0.001
Vessel Invasion (present vs. absent)	2.662 (1.672–4.236)	<0.001		
ADAMTS19 expression (high vs. low)	0.596 (0.373–0.953)	0.031		
S100A16 expression (high vs. low)	1.681 (1.068–2.647)	0.025	1.666 (1.052–2.638)	0.030

## Data Availability

All data are contained within the article or Supplementary Material.

## Supplementary Materials

The following are available online at https://www.mdpi.com/article/10.3390/biom11040561/s1, Figure S1: Pearson’s correlation coefficients in MEXPRESS showed that ADAMTS19 expression negatively correlated with promoter methylation; Figure S2: ADAMTS19 tended to affect the levels of E-cadherin and β-catenin measured by Western blot; Figure S3: Uncropped Western blots; Table S1: Distinct genes after ADAMTS19 overexpression; Figure S4: The overall survival based on WHO classification and Lauren’s classification.

## References

[1] Bray F., Ferlay J., Soerjomataram I., Siegel R.L., Torre L.A., Jemal A. (2018). Global cancer statistics 2018: GLOBOCAN es-timates of incidence and mortality worldwide for 36 cancers in 185 countries. CA: A Cancer J. Clin..

[2] Smyth E.C., Nilsson M., Grabsch H.I., van Grieken N.C., Lordick F. (2020). Gastric cancer. Lancet.

[3] Hamashima C. (2014). Current issues and future perspectives of gastric cancer screening. World J. Gastroentero..

[4] Kelwick R., Desanlis I., Wheeler G.N., Edwards D.R. (2015). The ADAMTS (A Disintegrin and Metalloproteinase with Throm-bospondin motifs) family. Genome Biol..

[5] Mead T.J., Apte S.S. (2018). ADAMTS proteins in human disorders. Matrix Biol..

[6] Cal S., López-Otín C. (2015). ADAMTS proteases and cancer. Matrix Biol..

[7] Binder M.J., McCoombe S., Williams E.D., McCulloch D.R., Ward A.C. (2017). The extracellular matrix in cancer progression: Role of hyalectan proteoglycans and ADAMTS enzymes. Cancer Lett..

[8] Li C., Luo X., Huang B., Wang X., Deng Y., Zhong Z. (2020). ADAMTS12 acts as a cancer promoter in colorectal cancer via activating the Wnt/β-catenin signaling pathway in vitro. Ann. Transl. Med..

[9] Binder M.J., McCoombe S., Williams E.D., McCulloch D.R., Ward A.C. (2020). ADAMTS-15 Has a Tumor Suppressor Role in Prostate Cancer. Biomolecules.

[10] Huang J., Sun Y., Chen H., Liao Y., Li S., Chen C., Yang Z. (2019). ADAMTS5 acts as a tumor suppressor by inhibiting migration, invasion and angiogenesis in human gastric cancer. Gastric Cancer.

[11] Jiang C., Zhou Y., Huang Y., Wang Y., Wang W., Kuai X. (2019). Overexpression of ADAMTS-2 in tumor cells and stroma is predictive of poor clinical prognosis in gastric cancer. Hum. Pathol..

[12] Alonso S., González B., Ruiz-Larroya T., Durán Domínguez M., Kato T., Matsunaga A., Suzuki K., Strongin A.Y., Gimènez-Bonafé P., Perucho M. (2015). Epigenetic inactivation of the extracellular matrix metallopeptidase ADAMTS19 gene and the metastatic spread in colorectal cancer. Clin. Epigenetics.

[13] Sturchler E., Cox J.A., Durussel I., Weibel M., Heizmann C.W. (2006). S100A16, a novel calcium-binding protein of the EF-hand superfamily. J. Biol. Chem..

[14] Babini E., Bertini I., Borsi V., Calderone V., Hu X., Luchinat C., Parigi G. (2011). Structural characterization of human S100A16, a low-affinity calcium binder. J. Biol. Inorg. Chem..

[15] Zhou W., Pan H., Xia T., Xue J., Cheng L., Fan P., Zhang Y., Zhu W., Xue Y., Liu X. (2014). Up-regulation of S100A16 expression promotes epithelial-mesenchymal transition via Notch1 pathway in breast cancer. J. Biomed. Sci..

[16] Fang D., Zhang C., Xu P., Liu Y., Mo X., Sun Q., Abdelatty A., Hu C., Xu H., Zhou G. (2021). S100A16 promotes metastasis and progression of pancreatic cancer through FGF19-mediated AKT and ERK1/2 pathways. Cell Biol. Toxicol..

[17] Lv H., Hou H., Lei H., Nie C., Chen B., Bie L., Han L., Chen X. (2020). MicroRNA-6884-5p Regulates the Proliferation, Invasion, and EMT of Gastric Cancer Cells by Directly Targeting S100A16. Oncol. Res..

[18] Camp R.L., Dolled-Filhart M., Rimm D.L. (2004). X-tile: A new bio-informatics tool for biomarker assessment and out-come-based cut-point optimization. Clin. Cancer Res..

[19] Huang D.W., Sherman B.T., Lempicki R.A. (2009). Systematic and integrative analysis of large gene lists using DAVID bioinformatics resources. Nat. Protoc..

[20] Huang D.W., Sherman B.T., Lempicki R.A. (2009). Bioinformatics enrichment tools: Paths toward the comprehensive functional analysis of large gene lists. Nucleic Acids Res..

[21] Jones P.A. (2012). Functions of DNA methylation: Islands, start sites, gene bodies and beyond. Nat. Rev. Genet..

[22] Koch A., De Meyer T., Jeschke J., Van Criekinge W. (2015). MEXPRESS: Visualizing expression, DNA methylation and clinical TCGA data. BMC Genom..

[23] Tanaka M., Ichikawa-Tomikawa N., Shishito N., Nishiura K., Miura T., Hozumi A., Chiba H., Yoshida S., Ohtake T., Sugino T. (2015). Co-expression of S100A14 and S100A16 correlates with a poor prognosis in human breast cancer and promotes cancer cell invasion. BMC Cancer.

[24] Chen D., Luo L., Liang C. (2018). Aberrant S100A16 expression might be an independent prognostic indicator of unfavorable survival in non-small cell lung adenocarcinoma. PLOS ONE.

[25] Zhang Q., Lenardo M.J., Baltimore D. (2017). 30 Years of NF-κB: A Blossoming of Relevance to Human Pathobiology. Cell.

[26] Li S., Lv M., Qiu S., Meng J., Liu W., Zuo J., Yang L. (2019). NF-κB p65 promotes ovarian cancer cell proliferation and migration via regulating mortalin. J. Cell Mol. Med..

[27] Echizen K., Horiuchi K., Aoki Y., Yamada Y., Minamoto T., Oshima H., Oshima M. (2019). NF-κB-induced NOX1 activation promotes gastric tumorigenesis through the expansion of SOX2-positive epithelial cells. Oncogene.

[28] Zhang Y., Huo F., Wei L., Gong C., Pan Y., Mou J., Pei D. (2017). PAK5-mediated phosphorylation and nuclear translocation of NF-κB-p65 promotes breast cancer cell proliferation in vitro and in vivo. J. Exp. Clin. Cancer Res..

