# Correction: Jiang et al. ADAMTS19 Suppresses Cell Migration and Invasion by Targeting S100A16 via the NF-κB Pathway in Human Gastric Cancer. *Biomolecules* 2021, *11*, 561

**DOI:** 10.3390/biom16010050

**Published:** 2025-12-29

**Authors:** Yingming Jiang, Xihu Yu, Yandong Zhao, Jintuan Huang, Tuoyang Li, Hao Chen, Junyi Zhou, Zhenze Huang, Zuli Yang

**Affiliations:** 1 Department of Gastrointestinal Surgery, the Sixth Affiliated Hospital, Sun Yat-sen University, Guangzhou 510275, China; 2 Guangdong Provincial Key Laboratory of Colorectal and Pelvic Floor Diseases, the Sixth Affiliated Hospital, Sun Yat-sen University, Guangzhou 510275, China; 3 Department of Pathology, the Sixth Affiliated Hospital, Sun Yat-sen University, Guangzhou 510275, China

In the original publication [1], the cell line sourcing information in Section 2.4 was incomplete. The original text stated that all cell lines “were obtained from the Type Culture Collection Cell Bank of the Chinese Academy of Sciences Committee (Shanghai, China)”, which did not accurately reflect the diverse sources from which the cell lines were acquired. Additionally, there was a typographical error in the cell line name (“BGC803” instead of “BGC823”).

**A correction has been made to Section 2.4:**

Seven human gastric cancer (GC) cell lines (MKN1, MKN45, MGC803, BGC823, HGC27, SGC7901, and AGS) and one human normal gastric mucosal cell (GES1) used in this study were obtained from certified and authenticated cell banks. Detailed source information is as follows: HGC27 (accession number: SCSP-5263), AGS (accession number: SCSP-5262) and SGC7901 (accession number: TCHu46) were obtained from the National Collection of Authenticated Cell Culture; MGC803 (accession number: CBP60485), BGC823 (accession number: CBP60477) and MKN1 (accession number: CBP60486) were obtained from ATCC; MKN45 (accession number: CVCL_0434) was obtained from JCRB Cell Bank. The human normal gastric mucosal cell GES1 was obtained from the Type Culture Collection Cell Bank of the Chinese Academy of Sciences Committee (Shanghai, China).

The authors state that the scientific conclusions are unaffected. This correction was approved by the Academic Editor. The original publication has also been updated.
