# Supplementary material for: ADAMTS19 Suppresses Cell Migration and Invasion by Targeting S100A16 via the NF-κB Pathway in Human Gastric Cancer
Source: Biomolecules. 2021 Apr 12;11(4):561. doi: 10.3390/biom11040561 (PMC8070242; doi:10.3390/biom11040561)
Supplement: Supplementary file 1 [file biomolecules-11-00561-s001.zip › Supplementary Files/Supplementary Figures and legends.pdf]

Supporting Information

ADAMTS19 suppresses cell migration and invasion by targeting S100A16 via the NF-κB pathway in human gastric cancer

Yingming Jiang[1,2,3], Xihu Yu[1,2,3], Yandong Zhao[1,2,3], Jintuan Huang[1,2,3], Tuoyang Li[1,2,3], Hao Chen[1,2,3], Junyi Zhou[1,2,3], Zhenze Huang[1,2,3], Zuli Yang[1,2,3]

Supplementary Figures

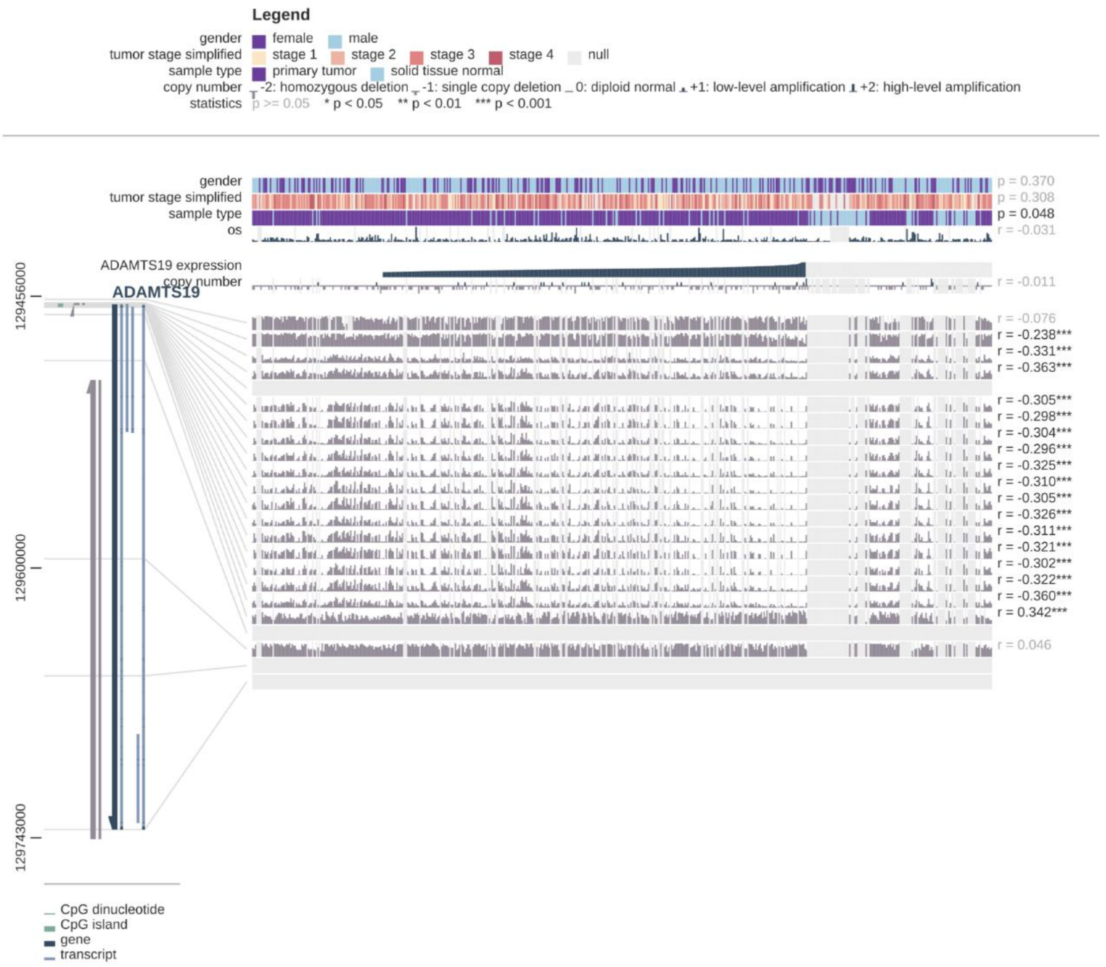

Figure S1. Pearson's correlation coefficients in MEXPRESS showed that ADAMTS19 expression negatively correlated with promoter methylation. *$P < 0.05$; **$P < 0.01$; ***$P < 0.001$.

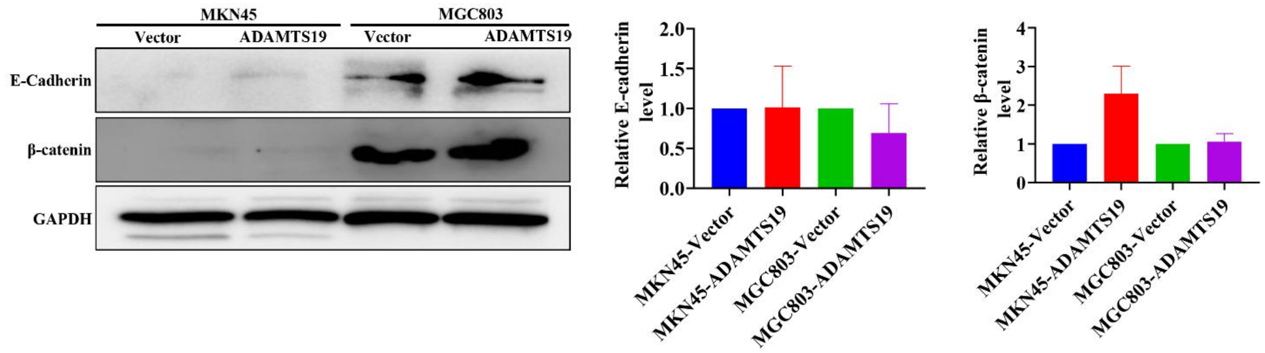

Figure S2. ADAMTS19 tended to affect the levels of E-cadherin and β-catenin measured by western blot.

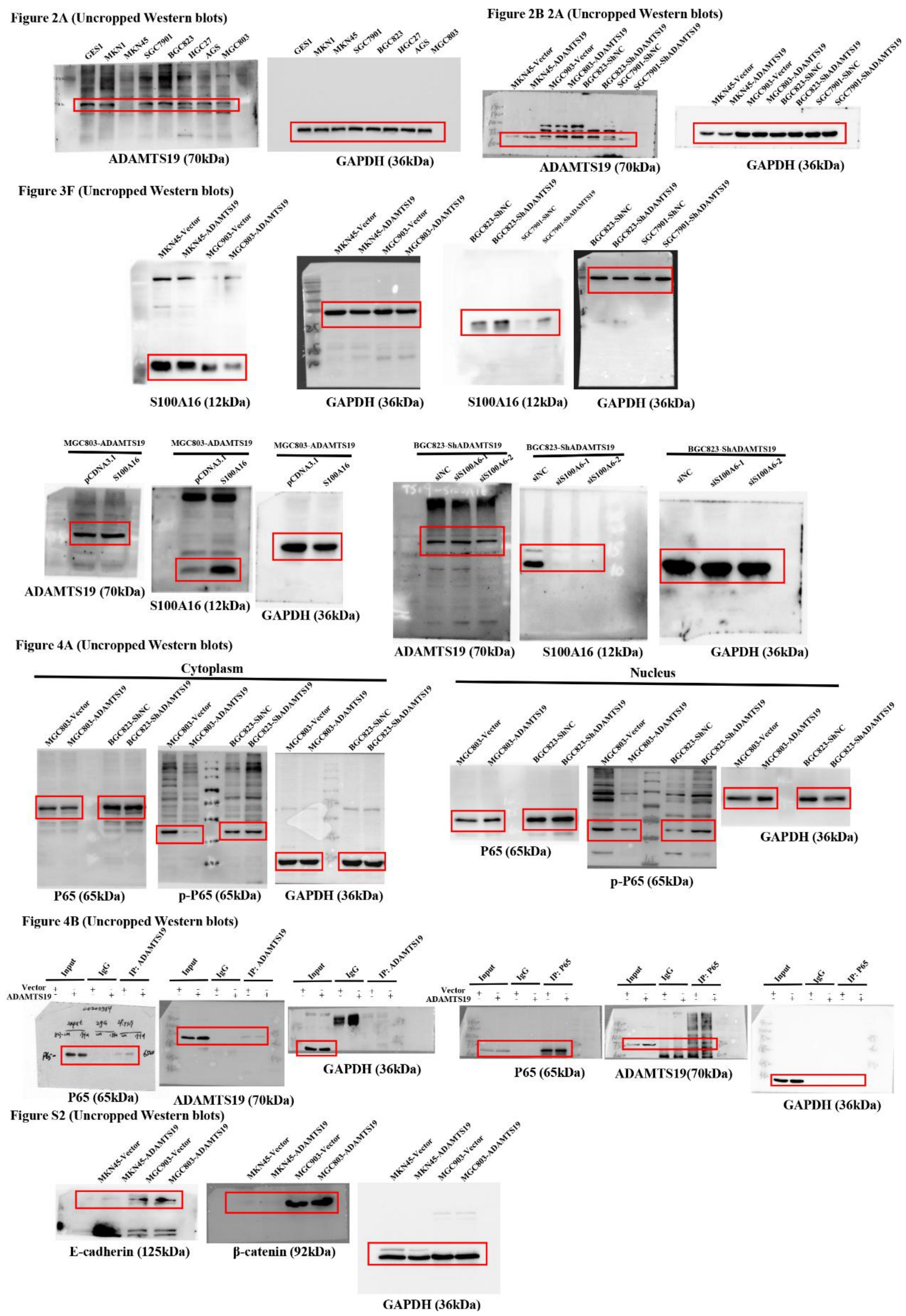

Figure S3. Uncropped Western blots.

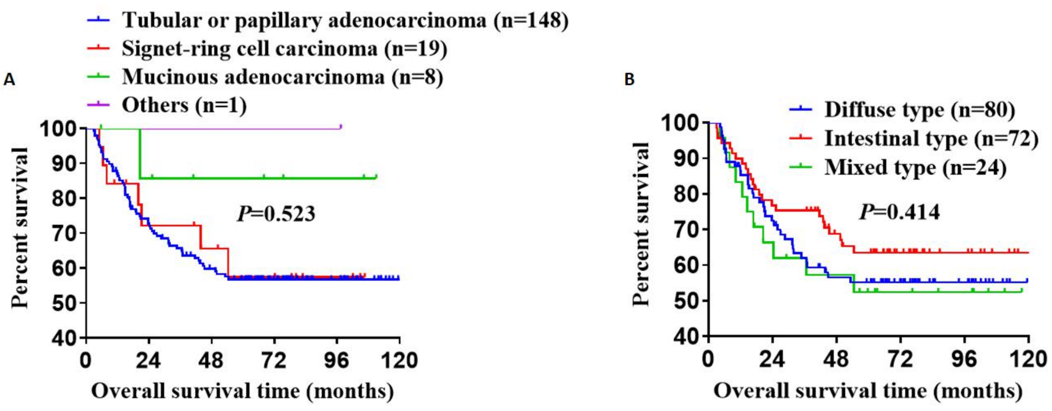

Figure S4. The Overall Survival based on WHO classification and Lauren's classification.
