# Supplementary figures and images for: ADAMTS19 Suppresses Cell Migration and Invasion by Targeting S100A16 via the NF-κB Pathway in Human Gastric Cancer
Source: Biomolecules. 2021 Apr 12;11(4):561. doi: 10.3390/biom11040561 (PMC8070242; doi:10.3390/biom11040561)

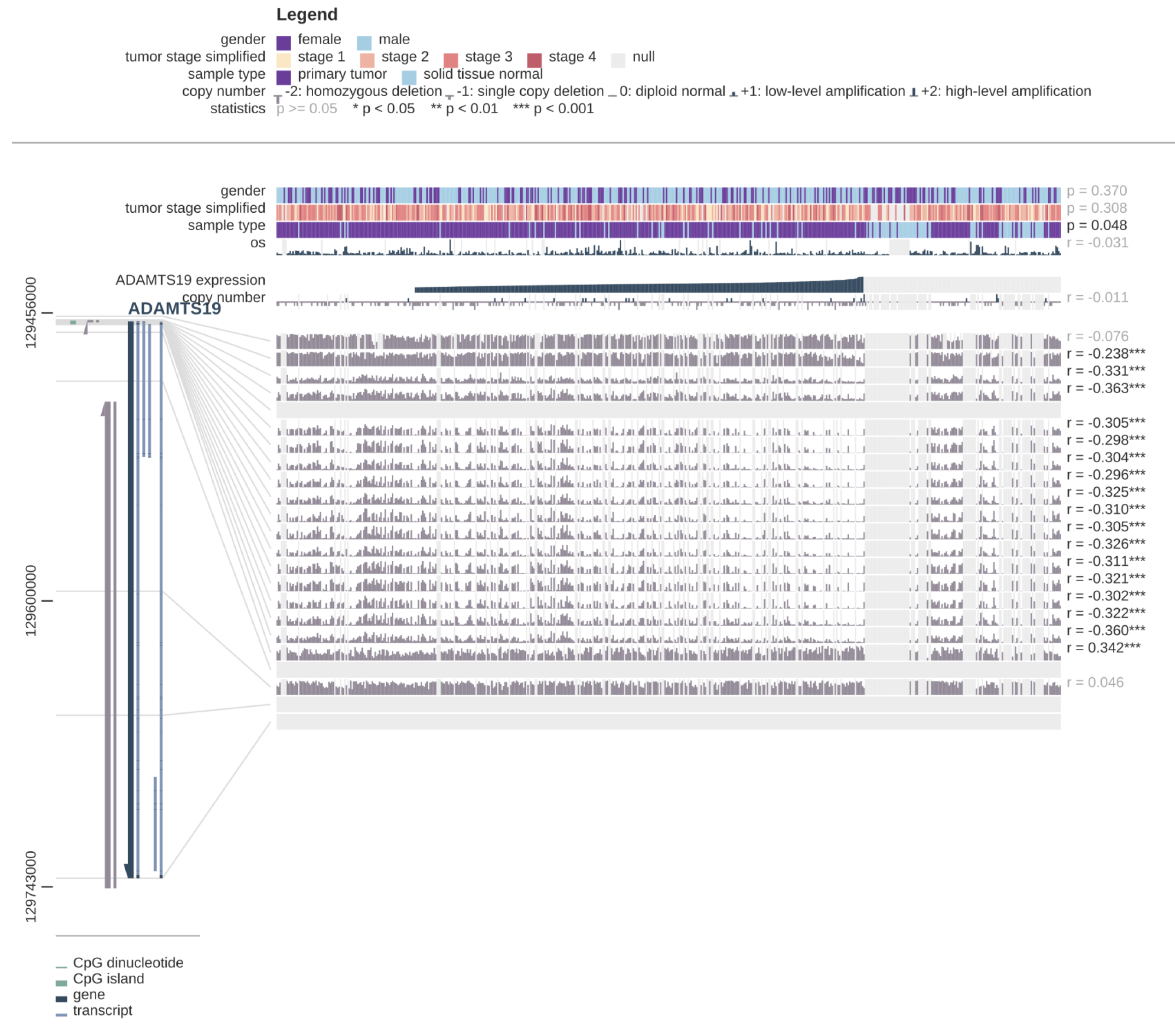

Supplement: Supplementary file 1 [file biomolecules-11-00561-s001.zip › Supplementary Files/Figure S1.tif]

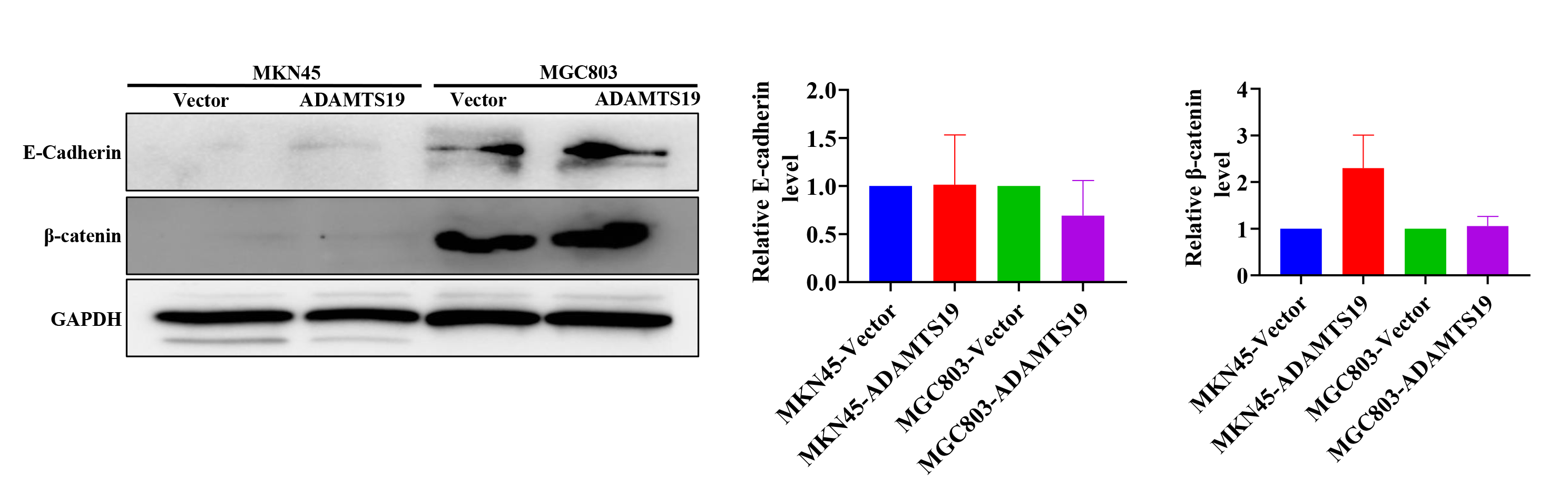

Supplement: Supplementary file 1 [file biomolecules-11-00561-s001.zip › Supplementary Files/Figure S2.tif]

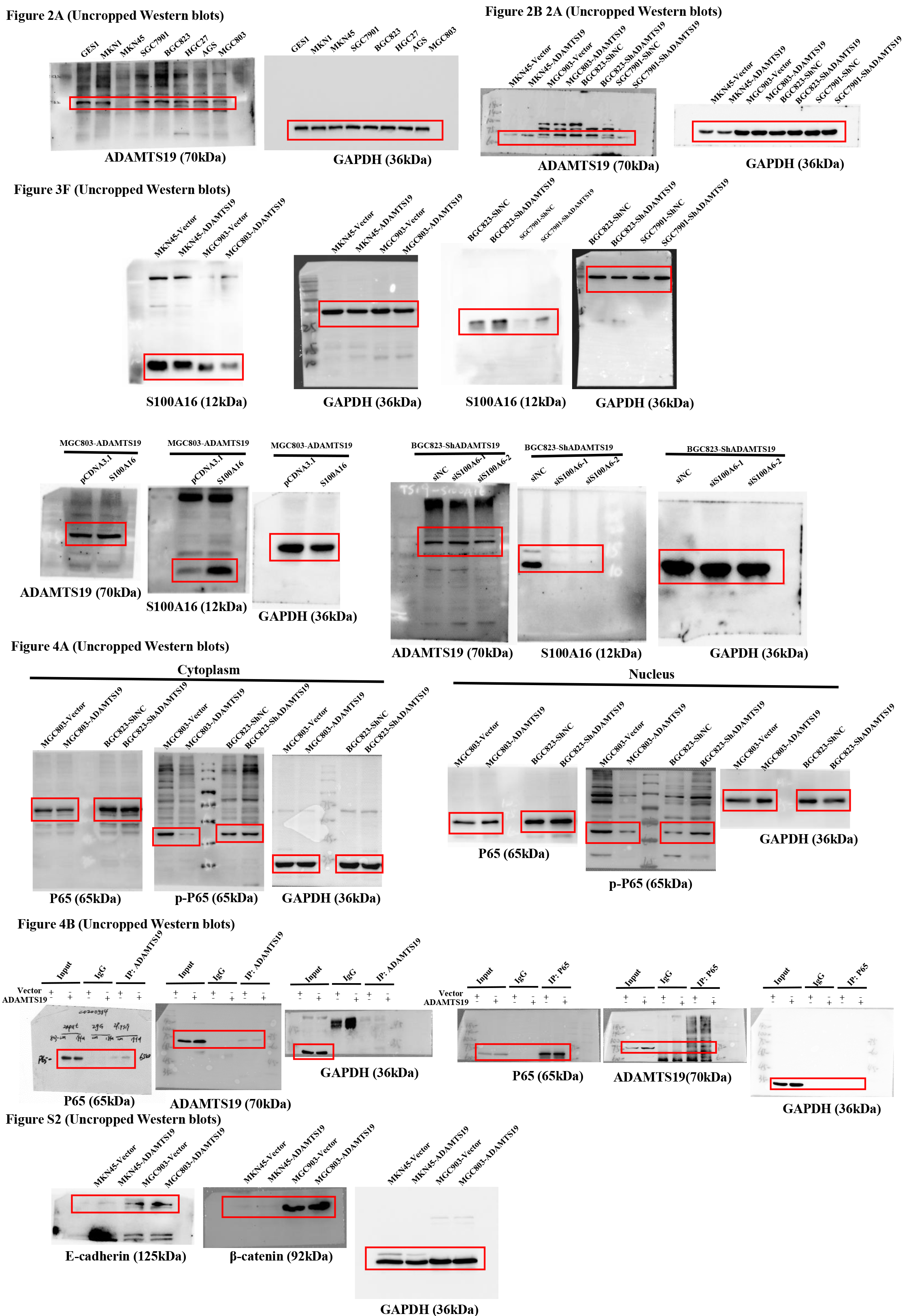

Supplement: Supplementary file 1 [file biomolecules-11-00561-s001.zip › Supplementary Files/Figure S3.tif]

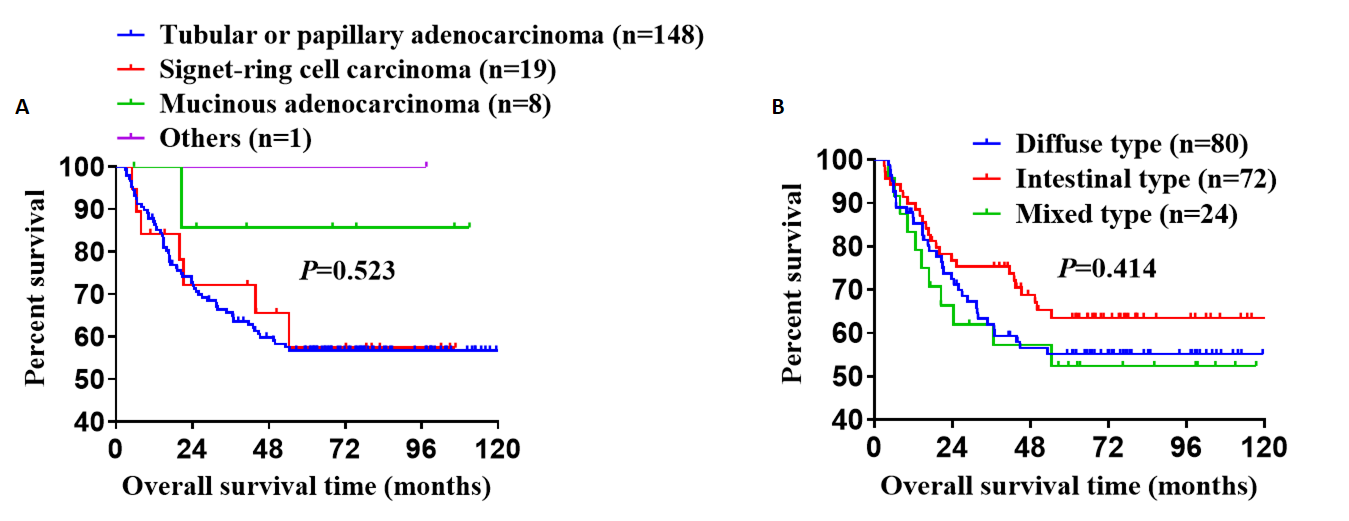

Supplement: Supplementary file 1 [file biomolecules-11-00561-s001.zip › Supplementary Files/Figure S4.bmp]
